# Occurrence of toxic metals and their selective pressure for antibiotic-resistant clinically relevant bacteria and antibiotic-resistant genes in river receiving systems under tropical conditions

**DOI:** 10.1007/s11356-021-17115-z

**Published:** 2021-11-05

**Authors:** Dhafer Mohammed M. Al Salah, Amandine Laffite, Periyasamy Sivalingam, John Poté

**Affiliations:** 1 Department F. A. Forel, Faculty of Sciences, Earth and Environmental Sciences, Institute F. A. Forel and Institute of Environmental Sciences, University of Geneva, Bd Carl-Vogt 66, CH-1211 Geneva 4, Switzerland; 2 King Abdulaziz City for Science and Technology, Joint Centers of Excellence Program, Prince Turki the 1st St, Riyadh, 11442 Saudi Arabia; 3 Postgraduate and Research Department of Microbiology, Jamal Mohamed College, Tamil Nadu, Tiruchirappalli, 620020 India

**Keywords:** Heavy metals, Sediments, Urban wastewater, Pollution, Antibiotic resistance, Public health

## Abstract

The co-occurrence of heavy metals, antibiotic-resistant bacteria (ARB) and antibiotic-resistance genes (ARGs) from hospital effluents spreading into the river receiving systems and evaluating associated risks are topics of scientific interest and still under-studied in developing countries under tropical conditions. To understand the selectors of the ARGs, we examined the occurrence of heavy metals (Cr, Co, Ni, Cu, Cd, Pb and Zn), associated ARB (β-lactam-resistant *Escherichia coli*, β-lactam-resistant Enterobacteriaceae, and carbapenem-resistant Enterobacteriaceae) and ARGs (*bla*_*OXA*_, *bla*_*CTX-M*_, *bla*_*IMP*_, *bla*_*TEM*_) in water and sediments from two sub-urban rivers receiving urban and hospital effluent waters in the Democratic Republic of the Congo (DRC). High abundances of ARB and ARGs were observed in all sediment samples. All the metal contents correlated negatively with grain size (− 0.94 ≤ *r* ≤  − 0.54, *p* < 0.05) except for Ni and positively with organic matter content and total copies of 16 s rRNA (0.42 ≤ *r* ≤ 0.79, *p* < 0.05), except for Ni and Zn. The metals had a significant positive correlation with the faecal indicator *Enterococcus* except for Ni and Cd (0.43 ≤ *r* ≤ 0.67, *p* < 0.05). Carbapenem-resistant Enterobacteriaceae correlated negatively with Zn (*r* =  − 0.44, *p* < 0.05) and positively with all the rest of toxic metals (0.58 ≤ *r* ≤ 1.0, *p* < 0.05). These results suggested that some metals had a great influence on the persistence of ARB and ARGs in sediments. Overall, this study strongly recommends the managing urban wastewater to preserve water resources used for human and agricultural purposes. Additionally, we recommend the utilizing biological indicators (faecal indicator bacteria, ARB, ARGs) when investigating urban wastewater pollutions.

**Supplementary Information:**

The online version contains supplementary material available at 10.1007/s11356-021-17115-z.

## Introduction

All human civilizations that sprang in the past have depended on and were structured around freshwater resources. Freshwater resources are a precious commodity and essential to human life and have been declared a human right by the United Nations General Assembly in Resolution 64/292 (Ki-moon and General 2010; Daily et al. 2012). However, the spread and dissemination of ARB and ARGs in the fresh water environment have become a global health concern and considered to be an emerging environmental pollutant (Sivalingam et al. 2020; Devarajan et al. 2016; Sanderson et al. 2016). Several factors other than antibiotics influence the spread and dissemination of ARGs in the environment (Devarajan et al. 2015). More worryingly, in developing countries, such as Sub-Saharan African countries, the discharge of raw sewage into rivers is common (Al Salah et al. 2020; Laffite et al. 2020, 2016). This practice deteriorates the quality of freshwater resources by contaminants of ARGs, heavy metals, antibiotics and ARB (Tripathi and Tripathi 2017, Ngweme et al. 2021). Toxic metals also adversely affect the environment and its flora which could disturb the balance of the impacted ecosystems (Poté et al. 2008); Haller et al. 2011). Intensive anthropogenic activities such as mining, burning of fossil fuels, agricultural practices, and discharge of industrial, urban or hospital effluents into the aquatic ecosystems are attributed to heavy metal pollution. Sediments receiving urban and hospital wastewaters under tropical conditions have been proven to contain unhealthy amounts of pathogens, ARB, ARGs, persistent organic pollutants (POPs) and heavy metals (Al Salah et al. 2019, Al Salah et al. 2020, Laffite et al. 2020, Suami et al. 2020, Anand et al. 2021, Ngweme et al. 2021). Although some bacteria have a natural ability to resist and biodegrade antibiotics, several factors favour the propagation of antibiotic resistance in microbes (Kummerer 2004, Tripathi and Cytryn 2017, Santos-Lopez et al. 2019, Baquero et al. 2021). The discharge of partially treated or untreated hospital effluents into aquatic environments complicates the issue further compared to treated wastewaters. Furthermore, abiotic factors such as heavy metals have been shown to influence ARGs’ co-selection and potential to spread in the environment. For instance, several previous studies reported co-selection of ARGs in the environment by heavy metals (Dickinson et al. 2019; Mazhar et al. 2020). Consequently, the positive correlations between heavy metals, ARB and ARGs were shown to have occurred in the environment (Laffite et al. 2020, 2016). Moreover, the low concentration of toxic metals has been shown to induce ARGs’ conjugative transfer (Zhang et al. 2018; Baker-Austin et al. 2006). Temperature, acidity, organic matter content and salinity of the soil/sediment influence metals’ absorption rate by plants, but generally, vegetables absorb the most metals followed by roots, cereals and then fruits (Gramlich et al. 2017; McLaughlin 2016). The discharge of effluent with a relatively high metal content presents a potential health risk via direct ingestion of these metals or the selective pressure for antibiotic resistance. For example, arsenic can disturb the metabolite of cells and the process of mitosis, Pb causes a spectrum of diseases from arthritis to kidney and brain damage, and Cd causes nephrotoxicity (Gordon and Quastel 1948; Martin and Griswold 2009; Castagnetto et al. 2002). Also, most heavy metals cause cancer (Martin and Griswold 2009; Järup 2003). Beyond the critical role of heavy metals in ARGs’ co-selection, disinfectants and pharmaceuticals beside antibiotics could promote natural transformation (Hassoun-Kheir et al. 2020). In the Democratic Republic of the Congo (DRC), the study site, Food and Agriculture Organization (FAO) supports urban gardeners through the Urban horticulture Project, which saw 122% increase in production in 5 years (Mutshail 2014). However, the environmental contamination of heavy metals in the freshwater resources in DRC has been considered an emerging threat to human and environmental health (Kilunga et al. 2017).

Although there are studies on ARB and ARGs in the urban river environment of DRC, most are restricted to hospital discharging points and no studies have yet been conducted to investigate the presence of heavy metals and correlating the presence of ARB and ARGs in this ecosystem (Al Salah et al. 2020). Therefore, the present study aims to determine heavy metals in the water and sediment of two river systems receiving urban and hospital effluent waters and the correlation with ARB and ARGs. The correlations will shed light on this river receiving environment for the selective pressure exerted by heavy metals on the accumulation of ARB and ARGs which has rarely been studied in a tropical system.

## Materials and methods

### Sampling site

According to our previous data (Al Salah et al. 2020), two river receiving systems were selected. The rivers drain through Kinshasa, the DRC capital city and receive effluent waters from two healthcare systems (a hospital and a clinic). These rivers serve as primary domestic use sources, fishing, bathing, drinking water supply and irrigation for urban agriculture, and they have a great socio-economic value. The sources of river contamination are different in both systems based on the detection of *Escherichia coli* upstream (Al Salah et al. 2020). In the system receiving effluent from the clinic, *E. coli* was detected upstream, suggesting multiple contamination sources. However, *E. coli* was not detected upstream in the river system receiving effluent from the hospital, suggesting the singularity of this system’s pollution. The sampling took place in May/September 2019 (dry season) and October/December 2019 (wet season). Two sampling campaigns were performed during each season. The water and surface sediments (0–3 cm layer) were collected in triplicate/season at the hospital/clinic effluent discharge points, and 100 m upstream and 100 m downstream. The surface sediments were chosen instead of deeper layers because it provides information on recent pollution and activities. The samples were kept at 4 °C in sterile clean plastic bottles before being shipped to the University of Geneva for analysis. The coordinates of the sampling sites are recorded in Supporting information (SI) (Table 1S).

### Water and sediment physicochemical parameters

The water physicochemical parameters including temperature (T), pH, electrical conductivity (EC) and dissolved oxygen (O_2_) values were measured in situ using a Multi 350i (WTW, Germany). A Laser Coulter LS-100 diffractometer (Beckman Coulter, Fullerton, CA, USA) was used to measure the sediment grain size including sand, silt and clay. For this analysis, the sediments were sonicated for 5 min in deionized water to consistently disperse the grains before being fed to the machine (Poté et al. 2008). The sediment organic matter (OM) was determined by loss on ignition (heating to 550 °C) for 1 h in Salvis oven (Salvis AG, Emmenbrücke, Luzern, Switzerland).

### Heavy metal analysis in water and sediment samples

Sediment samples were lyophilized and ground to fine powder before analysis. The digestion of sediment samples was performed as described by Poté et al. (2008) with minor modification. Briefly, around 1 g of the ground sediments was digested in 10 mL of 2 M analytical grade HNO_3_ in Teflon bombs at 100 °C for 16 h. The digestion product was subsequently centrifuged for 15 min at 4000 rpm and diluted 200 times in 1% HNO_3_ (Suprapur® 65%). For water analysis, 9.90 mL of water samples was filtered through a 0.22-μm syringe filter and acidified by the addition of 100 μL of Suprapur®, 65% HNO_3_.

Selected metals (Cr, Sc, Co, Ni, Cu, Cd, Pb and Zn) in digested sediment and water samples were then analysed using inductive coupled plasma-mass spectroscopy (Agilent 7700 × series ICP-MS, Santa Clara, USA). These metals were selected according to their toxicity occurrences in the study area (Laffite et al. 2016, 2020; Kilunga et al. 2017; Mwanamoki et al. 2015). A multi-element standard (Merck IV, KGaA-Darmstadt, Germany) solution at different concentrations (0, 0.2, 1, 5, 20 100, and 200 μg L^−1^) was used for calibration. The metal concentrations were expressed in ppm (mg kg^−1^ of dry sediment). The reference materials LKSD-3 and TMDA-51.4 for sediments and water, respectively, were used in order to verify the sensibility of the instrument and the reliability of the results. The average standard deviation of three replicates for ICP-MS analysis was below 5%, and the blanks remained undetected or below 1% of the sample signal, and the detection limit was less 0.009 ppm for all samples. Sc was selected to be used for geochemical normalization (Mwanamoki et al. 2015). The rest of the metals were selected for their toxicity and frequency of use in the studied area.

### Assessment of metal pollution degree in the sediments

Three indicators, enrichment factor (*EF*), geoaccumulation index (*I*_geo_) and pollution index (*PI*), which are important and subdivided into classes to highlight the degree of contamination were utilized to shed light on the amount of pollution in the studied region. These indicators were used to investigate the degree of sediment contamination according to Eqs. 1, 2 and 3 for *EF*, *I*_geo_ and *PI*, respectively and as described below:

1 $$EF=(metal/Sc)sample/(metal/Sc)background$$

*Metal:* measured concentration of each metal in the sediment samples.

*Sc*: concentration of Scandium in analysed sediment samples

2 $${I}_{geo}= {log}_{2}[({C}_{n}) / 1.5 ({B}_{n})]$$

*C*_*n*_: measured concentration of each metal in the sediment samples.

*B*_*n*_: concentration of the same metal in the geochemical background.

The values were calculated using the metal mean and lithogenic background values (Mwanamoki et al. 2015).

3 $$PI={C}_{i}/{S}_{i}$$

*C*_*i*_*:* measured concentration of each metal in the sediment.

*S*_*i*_: background value of each metal.

### Quantification of relevant bacteria and ARGs

*E. coli* and Enterobacteriaceae genus were selected because of their relevance to public health and their frequency of resistance to antibiotics, particularly against β-lactams and carbapenems, which are the most prescribed antibiotics and the last resort antibiotics against Gram-negative bacteria, respectively. The extraction of bacteria and DNA was carried out as previously stated (Al Salah et al. 2020). Briefly, 10 g of sediment was agitated horizontally for 1 h in a 50-mL solution of 0.2% sodium-hexa-metaphosphate followed by centrifugation for 15 min at 4000 rpm. The supernatant was utilized for bacterial quantification. The DNA was extracted from sediments using a PureLink Microbiome DNA purification kit (Invitrogen). The amount of copies of each gene was determined using quantitative PCR. The qPCR was performed using SensiFAST SYBR No-Rox kit in an Eco qPCR System (Illumina). The marker genes *bla*_*TEM*_, *bla*_*CTX-M*_, *bla*_*OXA*_, *bla*_*IMP*_ and 16S rRNA were targeted*.* We also investigated *bla*_*KPC*_ and *bla*_*SHV*_, but they were not detected anywhere in the investigated sites. The exact nature of each reaction of qPCR is reported in Table 2S (SI). Briefly, absolute quantification of target genes was performed using ten-fold dilutions of plasmids containing an ARG or the 16S RNA insert of known concentrations. Concentrations in samples were calculated by extrapolation compared to the standard curve. Any sample with a Ct value lower than that of the lowest of the dilutions was considered to be undetected.

### Statistical analysis

Rstudio version 1.2.5042 was used to generate statistical analysis (Team 2016). The package (Hmisc), which was developed by (Harrell, Harrell 2019), was used to generate a Pearson correlation matrix. The parameters of the matrix are metal content, organic matter content, mean grain size, which are reported in this study and total and antibiotic-resistant *E. coli* and Enterobacteriaceae and relevant bacterial markers and antibiotic-resistant genes in the sediments which were reported in our previous publication (Al Salah et al. 2020). The R package (ade4) was used to generate multiple principle component analysis (PCA) figures for a better visualization of the data (Dray and Dufour 2007) .

## Results and discussion

### Water physiochemical parameters

The results of water physicochemical parameters including T, pH, EC and O_2_ measured during the wet and dry seasons are presented in Table 1S (SI). The pH values from all the sampling sites were within the acceptable range set by the Food and Agriculture Organization (FAO) for water reuse. According to the pH readings, the waters tended to be slightly more acidic in the wet season in all the sites, except the hospital discharge point and upstream of it. These results can be explained due to the excessive rainfall during the wet season. In this study, no significant differences in temperature were noted between water samples, which could be attributed to tropical weather in Kinshasa. The electrical conductivity of most of the samples did not exceed 700 µS cm^−1^ set by the FAO (Ayers and Westcot 1985). These findings suggest that waters from the studied site might be used for irrigation. However, the electrical conductivity of the clinic in the dry season and downstream of the hospital in the wet season was above the 700 µS cm^−1^. The observed differences in the mean values of electrical conductivity can probably be attributed to high electrolytes present in the hospital effluents, which means that if these waters were to be used for irrigation, a degree of slight to moderate restriction must occur to ensure the safety of crops and farm workers (Ayers and Westcot 1985). It should be noted that local anthropogenic activities, climate and seasonality may also impact physical–chemical parameters and metal accumulation by plants. For instance, the pH level has an inversely proportional relation to plants’ metal uptake rate, while salinity and temperature have a directly proportional relationship (McLaughlin 2016; Gramlich et al. 2017).

The results of the sediments’ mean grain size and organic matter content are shown in SI (Table 3S). The sediments varied from silt at the clinic discharge point (mean grain size = 5.6 μm) to sand, upstream of the hospital discharge point (mean grain size = 212 μm). The organic matter content ranged from 0.12 to 7.66% with the highest recorded downstream of the hospital discharge point, followed by the hospital discharge point and the lowest upstream of the hospital discharge point. The observed organic matter content was typical for effluents with pollutants. High organic matter content begets lower metal uptake rates by crops (Gramlich et al. 2017).

### Metal content in water and sediments

Table 1 summarizes the average concentrations of metals in the water samples from the study sites in the wet and dry seasons. All the metals were below the maximum limit for irrigation water. They fell below the recommended values set by sediment quality guidelines (ANZECC 2000) and the FAO’s guidelines for water quality for agriculture (Ayers and Westcot 1985). However, the waters did not pass the microbiological test for irrigation water, as shown in our previous publication (Al Salah et al. 2020). The concentrations of metals in the water samples were well below the limit and had maximum values of (4.6), (1.4 × 10^−1^), (8.3), (2.52), (2.1 × 10^1^), (5.7 × 10^−1^), (5.3 × 10^−1^), and (5.0 × 10^1^) µg L^−1^ for Cr, Sc, Co, Ni, Cu, Cd, Pb and Zn, respectively. The highest and lowest values of Cr were in the hospital effluent during the wet season and upstream of the hospital effluent during the dry season, respectively. Co remained detectable in all the sites during both seasons with the highest value recorded at the hospital discharge point in the wet season and the lowest upstream of the hospital during the wet season as well. Ni was the least detectable in the sites and was only detectable at the hospital discharge point in both seasons and at the clinic discharge point in the dry season. The nickel concentrations were similar to a report in China and Egypt but lower than in Mexico and South Africa (Gutiérrez et al. 2008; Li and Zhang 2010; Olujimi et al. 2015; Osman and Kloas 2010). Cu was the highest at the hospital in the wet season and was not detectable in the same site during the dry season. Copper values were lower than those reported in Nigeria, Egypt, India and South Africa (Ashokkumar et al. 2009; Ohimain et al. 2008; Olujimi et al. 2015; Osman and Kloas 2010). The highest value of Cd was recorded upstream of the hospital discharge point during the wet season. The concentrations of Pb were not detectable during the dry season except at the clinic, and the highest value was recorded at the clinic discharge point during the wet season. The Pb concentrations were lower than those reported in South Africa and Nigeria (Ohimain et al. 2008; Olujimi et al. 2015). In both seasons, Zn remained detectable in all sites, and the highest value was recorded during the dry season downstream of the hospital discharge point.

**Table 1:** The average of metal concentrations in water during the wet and dry season in micrograms per litre

Site	Season	Cr	Sc	Co	Ni	Cu	Cd	Pb	Zn
Upstream Hospital	Dry	0.00	0.03	0.05	0.00	0.12	0.03	0.00	2.13
Hospital		0.27	0.13	1.09	1.00	0.00	0.00	0.00	10.96
Downstream Hospital		0.05	0.07	0.08	0.00	0.52	0.20	0.00	49.67
Upstream Clinic		0.10	0.08	0.16	0.00	0.30	0.00	0.00	14.46
Clinic		0.29	0.14	0.57	2.52	0.93	0.01	0.21	12.49
Downstream Clinic		0.12	0.08	0.13	0.00	0.57	0.00	0.00	8.02
Upstream Hospital	Wet	0.16	0.00	0.04	0.00	0.41	0.57	0.08	3.14
Hospital		4.45	0.00	8.26	2.52	21.03	0.02	0.47	5.86
Downstream Hospital		0.61	0.00	0.67	0.00	2.12	0.01	0.32	2.48
Upstream Clinic		0.05	0.06	0.10	0.00	0.21	0.01	0.00	8.30
Clinic		0.42	0.00	0.21	0.00	2.24	0.11	0.53	19.14
Downstream Clinic		0.09	0.07	0.10	0.00	0.55	0.10	0.00	28.85
FAO limits (Ayers and Westcot 1985)		100	NA	50	200	200	10	5000	2000

**Fig. 1:**
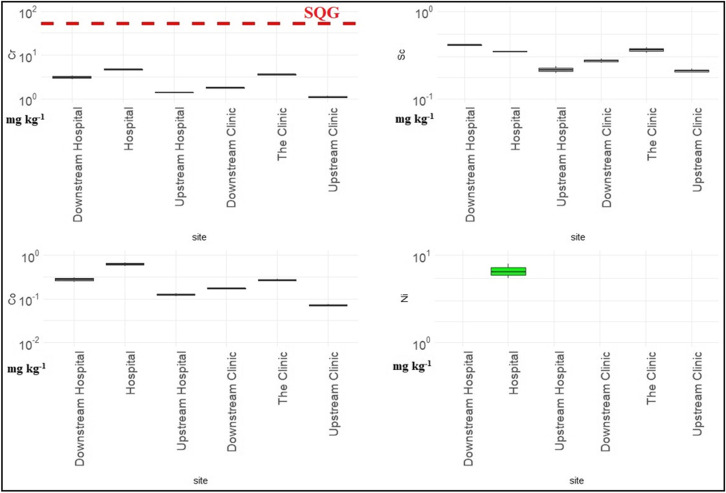
The metal content of Cr, Sc, Co and Ni in the sediments in mg kg^−1^ of dry weight in the log scale. The dashed line represents the limit set by the sediment quality guidelines (SQG)

**Fig. 2:**
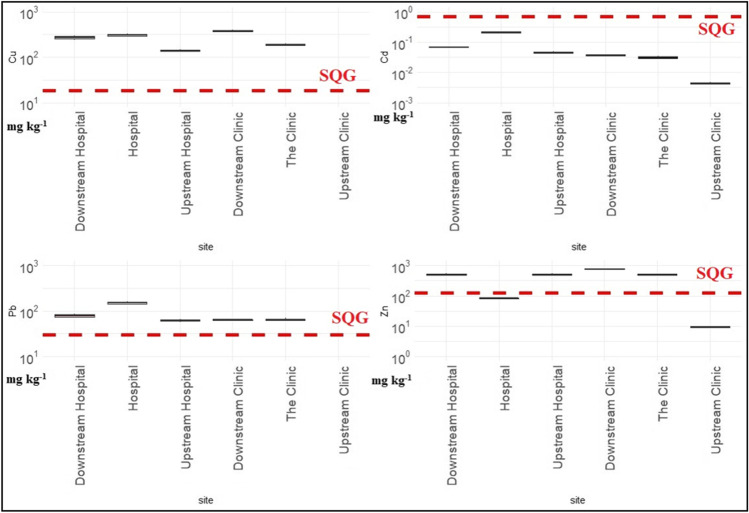
The metal content of Cd, Cu, Pb and Zn in the sediments in mg kg^−1^ of dry weight in the log scale. The dashed line represents the limit set by the sediment quality guidelines (SQG)

Figures 1 and 2 illustrate metal contents in the sediments and the dashed lines represent the sediment quality guidelines (SQG) (Environment 2001). In general, the concentrations of metals in the sediments were higher than in water which is in agreement with the literature (Shanbehzadeh et al. 2014; Salati and Moore 2010). The metal content in the sediment samples ranged between 1.09 and 4.62, 0.21 and 0.41, 0.07 and 0.62, 0 and 6.43, 0 and 377, 0.004 and 0.021, 0.038 and 151, and 9.27 and 758 mg kg^−1^ for Cr, Sc, Co, Ni, Cu, Cd, Pb and Zn, respectively. The levels of Cr, Sc, Co, Ni and Cd did not exceed the maximum limits SQGs (ANZECC 2000; Environment 2001). The hospital discharge point and upstream of the clinic had acceptable levels of Zn while the rest of the sites exceeded the limit. When comparing the study sites to three other studies under similar circumstances of receiving wastewater effluent, the study sites contained lower levels of Cd and Co than in Croatia, Nigeria and other rivers in Kinshasa but had higher levels of Cu and Pb. The Ni content was higher in Nigeria but lower than in Croatia and other sites in Kinshasa (Milaković et al. 2019; Ogwugwa et al. 2021; Laffite et al. 2016).

It is essential to highlight that there was not any significant difference between the discharge points and upstream of them in terms of metal content (*p* > 0.05). Therefore, we can conclude that the hospital and clinic in this study do not contribute significantly to heavy metal contamination in the area. The contamination could be attributed to other sources of contamination in particular diffuse sources such as uncontrolled landfills, agricultural runoff and various industries along these river banks. Additionally, the concentrations of fecal indicator bacteria have also been widely used to investigate freshwater pollution. It is highly recommended to utilize biological indicators instead of metal contents to investigate wastewater pollution in an area where pollution is ubiquitous because of their low cost, relative speed and convenience.

The metal pollution could accumulate and undergo biomagnification as it climbs the food web and present risk to public health. Besides, metal pollution presents a hazard to aquatic life forms and could negatively influence their otherwise stable ecosystems (Singh and Kalamdhad 2011).

### Assessment of metal pollution degree in the sediments

The background data was obtained from a previous publication and the authors chose Lake Mavallee in Kinshasa to serve as a background because of its pristine nature (Mwanamoki et al. 2015). Table 2 presents the enrichment factor values based on the formula mentioned above in the “Materials and methods”. Cr showed no enrichment to minor enrichment in all the sites. Co exhibited a similar pattern to Cr except at the hospital discharge point where Co showed moderate enrichment. Ni did not show any enrichment except moderately at the hospital discharge point. Cu and Pb showed extremely severe enrichment at all the sites except upstream of the clinic which had no enrichment. Cd enrichment degree varied from no enrichment to severe enrichment at the hospital discharge point. Zn underwent severe enrichment upstream of the clinic and extremely severe enrichment in all the other sites.

**Table 2:**
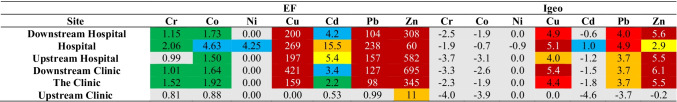

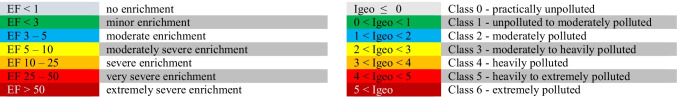
Classification of elemental enrichment and pollution based on the *EF* and geoaccumulation index. The key to the color-coding is below the table

The degree of pollution exhibited in Table 2 is based on the geo accumulation index. Upstream of the clinic discharge point remained pollution-free. All the sites had class 0 (practically unpolluted) for Cr, Co, Ni and Cd pollution except for Cd at the hospital discharge point with class 2 (moderately polluted). The Cu pollution was classified as class 4 (heavily polluted) upstream of the hospital, as class 5 (heavily to extremely polluted) downstream of the hospital and at the clinic discharge point and as class 6 (extremely polluted) at hospital discharge point and downstream of the clinic. Pb pollution was designated as class 4 upstream of the hospital, downstream of the clinic and the clinic’s discharge point and as class 5 discharge point of the hospital and downstream of it. Zn pollution scored class 6 in all the sites except at the hospital discharge point, which scored class 3 (moderately to heavily polluted), and upstream of the clinic which was unpolluted. The results highlight the high metal concentrations in all sampling sites, reaching values (in mg kg^−1^) of Cu (140–377), Pb (62–151) and Zn (83–758). According to the enrichment factor (*EF*), results showed extremely severe enrichment of Cu (159 ≤ *EF* ≤ 421), Pb (98 ≤ *EF* ≤ 238) and Zn (60 ≤ *EF* ≤ Zn) and extreme pollution as indicated by high geoaccumulation indices (*I*_geo_), which were (4.0 ≤ *I*_geo_ ≤ 5.4), (3.7 ≤ *I*_geo_ ≤ 4.9) and (2.9 ≤ *I*_geo_ ≤ 6.1), for Cu, Pb and Zn, respectively.

When comparing the *EF* and *I*_geo_ scores with rivers receiving wastewater effluent, our study sites scored higher enrichment and pollution of Cu, Pb and Zn but scored lower scores for Cd, Cr and Ni than in other urban rivers under tropical conditions (Hanif et al. 2016; Kilunga et al. 2017; Salati and Moore 2010).

The *PI* results (Table 3) concurred with the *I*_geo_ and indicated very strong levels of Cu, Pb and Zn pollution in all the sites except for upstream of the hospital and high level of Cd pollution only at the hospital discharge point. When comparing the *EF* and *I*_geo_ scores with rivers receiving wastewater effluent, our study sites scored higher enrichment and pollution of Cu, Pb and Zn than in other rivers in Kinshasa, DRC, Iran and Pakistan but scored lower scores for Cd, Cr and Ni (Hanif et al. 2016; Kilunga et al. 2017; Salati and Moore 2010).

**Table 3:** Classification of elemental pollution based on the pollution index

Site	Cr	Co	Ni	Cu	Cd	Pb	Zn
Downstream hospital	0	0	0	**46**	1	**24**	**71**
Hospital	0	1	1	**52**	*3*	**46**	**12**
Upstream hospital	0	0	0	**24**	1	**19**	**70**
Downstream clinic	0	0	0	**64**	1	**19**	**106**
Clinic	0	0	0	**32**	0	**20**	**70**
Upstream clinic	0	0	0	0	0	0	1

*PI* values are classified as the following: not polluted (*PI* < 1), low level of pollution (1 ≥ *PI* < 2), moderate level of pollution (2 ≥ *PI* < 3) assigned in italics, strong level of pollution (3 ≥ *PI* < 5) or very strong level of pollution (*PI* > 5) assigned in bold

### Quantification of relevant bacteria, 16 s rDNA, ARGs and bacterial markers

**Fig. 3:**
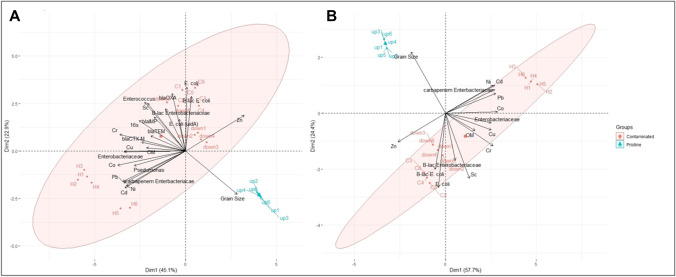
Principle component analyses **A** based on all biological and physical parameters and **B** based only on indicator bacteria, ARB and metals. H stands for hospital, C for clinic, up for upstream and down for downstream

The abundances of total and ARB indicators in water and sediments are illustrated in Fig. 1S and 2S (Supporting information (SI)), respectively. The 16S rRNA was quantified per gram of dry sediment (Fig. 3S), and bacterial markers and ARGs were expressed in relation to the total copies of 16 s rDNA, as shown in Figs. 4S and 5S (SI). We also investigated *bla*_*KPC*_ and *bla*_*SHV*_, but they were not detected anywhere in the investigated sites. This section has been discussed in depth previously (Al Salah et al. 2020).

In the dry season, the amounts of cultivable total and antibiotic-resistant *E. coli* and *Enterbacteriaceae* from the sediment were generally lower than the wet season, which is in agreement with a study performed in Nigeria (Achudume and Olawale 2009). However, the exact opposite is true for the water. The abundances in water were higher during the dry season, which could be attributed to the dilution by runoff as concluded by studies conducted under similar tropical conditions in Vietnam and India (Hoa et al. 2011; Pathak et al. 1993). The most widespread of ARGs was *bla*_*CTX-M*_ (Canton et al. 2012). Hospitals play a crucial role in the widespread of ARB and ARGs (Rodriguez-Mozaz et al. 2015; Graham et al. 2011; Xu et al. 2015; Calero-Caceres et al. 2017).

### Statistical analysis and correlation between metal and biological parameters

Several analysed parameters were used to construct a Pearson correlation matrix of which two were physical characteristics of the sediments (grain size and organic content), eight were metal contents in the sediments, and 13 were biological indicators (four relevant bacterial markers, four ARGs, and five total and antibiotic-resistant indicators), as shown in Table 4. The total 16S rRNA, *E. coli* (*uidA*)*, Pseudomonas*, *Enterococcus* and ARGs were quantified using quantitative polymerase reaction (qPCR), and β-lactam, carbapenem-resistant and total Enterobacteriaceae and *E. coli* were quantified using culture-based methods, as described and reported in our previous publication (Al Salah et al. 2020).

**Table 4:** Pearson correlation matrix between metal content, physicochemical parameters and biologically relevant bacteria and genes. (*) means significant at the 0.05 level. (**) means significant at the 0.01 level

	Cr	Co	Ni	Cu	Cd	Pb	Zn
OM	0.50*	0.54**	0.35	0.79**	0.58**	0.64**	0.29
Grain size	− 0.90**	− 0.70**	− 0.34	− 0.93**	− 0.54**	− 0.79**	− 0.66**
16 s	0.61**	0.52**	0.35	0.55**	0.42*	0.54**	0.26
*E. coli* (*uidA*)	0.05	− 0.03	− 0.22	0.30	− 0.04	0.07	0.45*
*Enterococcus*	0.62**	0.43*	0.16	0.62**	0.30	0.51*	0.53**
*Pseudomonas*	0.51*	0.65**	0.69**	0.42*	0.67**	0.60**	− 0.30
*bla*_*OXA*_	0.39	0.12	− 0.17	0.34	− 0.06	0.19	0.65**
*bla*_*CTX-M*_	0.48*	0.51*	0.44*	0.48*	0.50*	0.52**	0.01
*bla*_*IMP*_	0.53**	0.36	0.21	0.28	0.19	0.34	0.25
*bla*_*TEM*_	0.32	0.34	0.25	0.43*	0.35	0.38	0.11
*E. coli*	0.24	− 0.09	− 0.49*	0.45*	− 0.24	0.08	0.99**
Enterobacteriaceae	0.89**	0.90**	0.83**	0.64**	0.81**	0.85**	− 0.09
B-lactam-resistant *E. coli*	0.22	− 0.12	− 0.33	− 0.01	− 0.34	− 0.10	0.56**
B-lactam-resistant Enterobacteriaceae	0.53**	0.27	0.08	0.23	0.05	0.26	0.39
Carbapenem-resistant Enterobacteriaceae	0.73**	0.93**	1.00**	0.58**	0.96**	0.85**	− 0.44*
Cr		0.92**	0.69**	0.91**	0.81**	0.95**	0.28
Co			0.91**	0.84**	0.97**	0.99**	− 0.07
Ni				0.56**	0.96**	0.83**	− 0.48*
Cu					0.76**	0.92**	0.43*
Cd						0.94**	− 0.26
Pb							0.08
Zn							

Cr correlated at the 0.01 *p*-level negatively with grain size and positively with the total 16S rRNA, *Enterococcus*, *bla*_*IMP*_, all the metals except for Zn, β-lactam, carbapenem-resistant and total Enterobacteriaceae. Cr also correlated positively at the 0.05 level with *Pseudomonas, bla*_*CTX-M*_* a*nd organic matter content. Co correlated at the 0.01 level negatively with grain size and positively with 16S rRNA, *Pseudomonas*, organic matter, Ni, Cu, Cd, Pb, carbapenem-resistant and total Enterobacteriaceae. Co also correlated positively at the 0.05 level with *Enterococcus* and *bla*_*CTX-M*_. Ni correlated positively at the 0.01 level with *Pseudomonas*, Cu, Cd, Pb, carbapenem-resistant and total Enterobacteriaceae. Ni also correlated at the 0.05 level positively with *bla*_*CTX-M*_ and negatively with Zn and *E. coli.* Cu correlated at the 0.01 level negatively with grain size and positively with 16S rRNA, *Enterococcus*, organic matter, Cd, Pb, carbapenem-resistant and total Enterobacteriaceae. Cu also correlated positively at the 0.05 level with *Pseudomonas*, *bla*_*CTX-M*_, *bla*_*TEM*_, Zn and *E. coli.* Cd correlated at the 0.01 level negatively with grain size and positively with 16S rRNA, *Pseudomonas*, organic matter, Pb, carbapenem-resistant and total Enterobacteriaceae. Cd also correlated positively at the 0.05 level with 16S rRNA and *bla*_*CTX-M*_. Pb correlated at the 0.01 level negatively with grain size and positively with 16S rRNA, *Pseudomonas*, *bla*_*CTX-M*_, organic matter, carbapenem-resistant and total Enterobacteriaceae. Pb also correlated with *Enterococcus* at the 0.05 level. Zn correlated at the 0.01 level negatively with grain size and positively with *Enterococcus*, *bla*_*OXA*_, β-lactam-resistant and total *E. coli*. Zn also correlated at the 0.05 level positively with *E. coli* (*uidA*) and negatively with carbapenem-resistant Enterobacteriaceae.

Three PCA were performed. The first PCA utilized all the biological and metal parameters, which results from two distinguished clusters based on a total variance of 68% as displayed in Fig. 3A. Similarly, the second PCA utilized only indicator bacteria, ARB and metals, which also displays two distinct clusters based on a total variance of 82.1%, as shown in Fig. 3B. However, the third PCA was performed based only on the metal content and resulted in overlapping clusters, as shown in supplementary data (Fig. 6S), which support the previous hypothesis that utilizing biological indicators is a better approach to monitor pollution from wastewaters in the developing world than metal content. All the metals except for Zn correlated positively with one another which suggest a similar contamination source or a similar transport and retention pathway due to their positive charge (Karathanasis 1999; Naidu et al. 1994; Naidu et al. 1998; Sherene 2010). In general, the metals correlated positively with organic matter content and negatively with grain size. All of the studied metals correlated positively with at least one ARG, which could be attributed to the expression of metal resistance by those genes which have been reported extensively in the literature (Knapp et al. 2011; Laffite et al. 2016; Imran et al. 2019; Nguyen et al. 2019; Di Cesare et al. 2016).

## Conclusion

Each of the studied metals correlated significantly and positively with at least one ARG, suggesting the selection pressure of metals to antibiotic resistance. The levels of enrichment were extremely severe for Cu, Pb and Zn. However, the contribution of hospital effluent to the metal pollution was not significant in these study sites because of the lack of infrastructure and random unauthorized landfills and littering, which is ubiquitous in Kinshasa (DRC). Thus, this study showed varying influence on the ARG co-selection by river receiving systems. Therefore, we highly recommend utilizing biological indicators when investigating pollution in a city that lacks adequate infrastructure. Biological indicators are faster, cheaper and more convenient than metal content analysis. This is more important in countries where environmental regulations are often inadequate, and infrastructure is lacking. The findings can be helpful for the management of landfills and the establishment of adequate infrastructure to limit the spread of the contaminants to freshwater environments. This study will provide baseline information for the parameters relevant for the spreading of ARB, which will be an important measure to design strategies to control the metals, resistant bacteria and ARGs in the environment.

## Supplementary Information

Below is the link to the electronic supplementary material.

Supplementary file1 (DOCX 1174 kb)
