# Supplementary material for: Occurrence of toxic metals and their selective pressure for antibiotic-resistant clinically relevant bacteria and antibiotic-resistant genes in river receiving systems under tropical conditions
Source: Environ Sci Pollut Res Int. 2021 Nov 5;29(14):20530–41. doi: 10.1007/s11356-021-17115-z (PMC8898216; doi:10.1007/s11356-021-17115-z)
Supplement: Supplementary file 1 — Supplementary file1 (DOCX 1174 kb) [file 11356_2021_17115_MOESM1_ESM.docx]

**Supporting Information (SI)**

Occurrence of toxic metals and their selective pressure for antibiotic resistant clinically relevant bacteria and antibiotic resistant genes in river receiving systems under tropical conditions

Dhafer Mohammed M. Al Salah^1,2^, Amandine Laffite^1^, Periyasamy Sivalingam^3^, John Poté^1,3^^[[1]](#footnote-1)^*

*^1^University of Geneva, Faculty of Sciences, Earth and Environmental Sciences, Institute F. A. Forel and Institute of Environmental Sciences, Bd Carl-Vogt 66, CH-1211 Geneva 4*

*^2^King Abdulaziz City for Science and Technology, Joint Centers of Excellence Program, Prince Turki the 1^st^ st, Riyadh 11442, Saudi Arabia*

*^3^Postgraduate and Research Department of Microbiology, Jamal Mohamed College, Tiruchirappalli 620020, Tamil Nadu, India*

No of Tables = 3 (Table 1S - 3S)

No of Figures = 6 (Figure 1S- 6S)

**Table Caption**

Table 1S. Coordinates of sampling sites, their corresponding temperature, pH, electrical conductivity, and dissolved oxygen content

Table 2S. Quantitative PCR Primer Pairs for the Quantification of Genes of Interest, amplification efficiencies, and their respective size and annealing temperature

Table 3S. Types of sediments, grain size, organic matter content, and their clay, silt, and sand composition

**Table 1S.**

| **Season** | **Sampling site** | **Latitude** | **Longitude** | **pH** | **T (°C)** | **EC**  **(µS cm^-1^)** | **Dissolved O_2_ (mg/L)** |
| --- | --- | --- | --- | --- | --- | --- | --- |
| **Dry** | Upstream Hospital | 04⁰22᾿52.0ʺ | 015⁰17᾿30.5ʺ | 7.09 | 29.7 | 228.8 | 0.78 |
|  | Hospital | 04⁰22᾿56.8ʺ | 015⁰17’ 34.9ʺ | 7.35 | 25.3 | 673 | 1.88 |
|  | Downstream Hospital | 04⁰22᾿58.1ʺ | 015⁰17᾿37.3ʺ | 7.95 | 28.1 | 245 | 1.04 |
|  | Upstream Clinic | 04⁰23᾿42.3ʺ | 015⁰18᾿24.1ʺ | 7.14 | 27.1 | 551 | 1.44 |
|  | Clinic | 04⁰24᾿41.5ʺ | 015⁰18᾿8.9’’ | 7.01 | 29.4 | 997 | 1.02 |
|  | Downstream Clinic | 04⁰23᾿42.2ʺ | 015⁰18᾿25.0ʺ | 7.4 | 26 | 688 | 0.88 |
| **Wet** | Upstream Hospital | 04⁰22᾿52.0ʺ | 015⁰17᾿30.5ʺ | 7.5 | 28 | 204 | 3.7 |
|  | Hospital | 04⁰22᾿56.8ʺ | 015⁰17’ 34.9ʺ | 7.54 | 26.9 | 459 | 0.38 |
|  | Downstream Hospital | 04⁰22᾿58.1ʺ | 015⁰17᾿37.3ʺ | 6.9 | 27.1 | 907 | 1.98 |
|  | Upstream Clinic | 04⁰23᾿42.3ʺ | 015⁰18᾿24.1ʺ | 6.88 | 29.2 | 428 | 0.71 |
|  | Clinic | 04⁰24᾿41.5ʺ | 015⁰18᾿8.9’’ | 6.1 | 30 | 431 | 1.45 |
|  | Downstream Clinic | 04⁰23᾿42.2ʺ | 015⁰18᾿25.0ʺ | 6.68 | 29.1 | 441 | 0.38 |

**Table 2S.**

| **Target Gene** | **Primer** | **Sequence** | **Size of Target** | **Tm** | **R^2^** | **Amplification efficiency (%)** | **Reference** |
| --- | --- | --- | --- | --- | --- | --- | --- |
| **16s** | 338 F | ACTCCTACGGGAGGCAGCAG | 197 | 62 | 0.99 | 107 | (Ovreas et al. 1997) |
|  | 518 R | ATTACCGCGGCTGCTGG |  |  |  |  |  |
| **E. coli (UidA)** | Uida 405 F | CAACGAACTGAACTGGCAGA | 121 | 60 | 0.99 | 93 | (Chern et al. 2011) |
|  | Uida 405 R | CATTACGCTGCGATGGAT |  |  |  |  |  |
| **Pseudomonas spp.** | Pse435F | ACTTTAAGTTGGGAGGAAGGG | 251 | 62 | 0.99 | 96 | (Bergmark et al. 2012) |
|  | Pse686R | ACACAGGAAATTCCACCACCC |  |  |  |  |  |
| **Enterococcus** | Ent376F | GGACGMAAGTCTGACCGA | 220 | 62 | 0.99 | 92 | (Ryu et al. 2013) |
|  | Ent578R | TTAAGAAACCGCCTGCGC |  |  |  |  |  |
| **βla** _IMP_ | IMP-F | AAGTTAGTCA(A⁄C)TTGGTTTGTGGAGC | 269 | 58 | 0.99 | 91 | (Bisiklis et al. 2007) |
|  | IMP-R | CAAACCACTACGTTATCT(G ⁄T)GAGTGTG |  |  |  |  |  |
| **βla** _TEM_ | TEM-F | GCKGCCAACTTACTTCTGACAACG | 247 | 61 | 0.98 | 109 | (Sidrach-Cardona et al. 2014) |
|  | TEM-R | CTTTATCCGCCTCCATCCAGTCTA |  |  |  |  |  |
| **βla** _OXA-48_ | BlaOXA F | GCGTGGTTAAGGATGAACAC | 438 | 61 | 0.99 | 91 | (Poirel et al. 2011) |
|  | BlaOXA R | CATCAAGTTCAACCCAACCG |  |  |  |  |  |
| **βla** _CTX-M_ | blaCTX-M F | ATTCCRGGCGAYCCGCGTGATACC | 227 | 65 | 0.99 | 92 | (Fujita et al. 2011) |
|  | blaCTX-M R | ACCGCGATATCGTTGGTGGTGCCAT |  |  |  |  |  |

**Table 3S.**

| **Sampling site** | **Clay**  **(%)** | **Silt**  **(%)** | **Sand**  **(%)** | **Type of sediment** | **Mean grain size (μm)** | **Organic matter**  **(%)** |
| --- | --- | --- | --- | --- | --- | --- |
| **Upstream Hospital** | 0 | 2.4 | 97.6 | sand | 212 | 0.12 |
| **Hospital** | 1.1 | 45.8 | 53.1 | sandy loam | 56.4 | 3.52 |
| **Downstream Hospital** | 0.9 | 31 | 68.1 | sandy loam | 81.3 | 7.66 |
| **Upstream Clinic** | 1.63 | 13 | 85.37 | loamy sand | 97.3 | 1.89 |
| **Clinic** | 7.7 | 92.3 | 0 | silt | 5.6 | 1.75 |
| **Downstream Clinic** | 0.81 | 7.53 | 91.66 | sand | 171.8 | 1.46 |

**Figure Caption**

**Figure 1S**. The abundances of cultivable *E. coli*, Enterobacteriaceae (Ent), β-lactam resistant *E. coli* (B-lac *E. coli*) and Enterobacteriaceae (B-lac Ent), and carbapenem resistant *E. coli* (Carb E. coli) and Enterobacteriaceae (CRE) per mL of surface water in the wet and dry seasons

**Figure 2S**. The abundances of cultivable *E. coli*, Enterobacteriaceae (Ent), β-lactam resistant *E. coli* (B-lac E. coli) and Enterobacteriaceae (B-lac Ent), and carbapenem resistant *E. coli* (Carb E. coli) and Enterobacteriaceae (CRE) per gram of dry sediment in the wet and dry seasons

**Figure 3S**. Absolute abundances of 16S rRNA per gram of dry sediment in the wet and dry seasons

**Figure 4S**. Relative abundances of bacterial markers expressed as copies per copy of 16S rRNA in sediment in the wet and dry seasons

**Figure 5S**. Relative abundances of β-lactam resistant genes expressed copies per copy of 16S rRNA in the wet and dry seasons

**Figure 6S.** PCA analysis based only on the metal contents in the sediments. Up H stands for hospital upstream, H for hospital, down H for hospital downstream, up C for clinic upstream, C for clinic, and down C for clinic downstream.

**Figure 1S.**


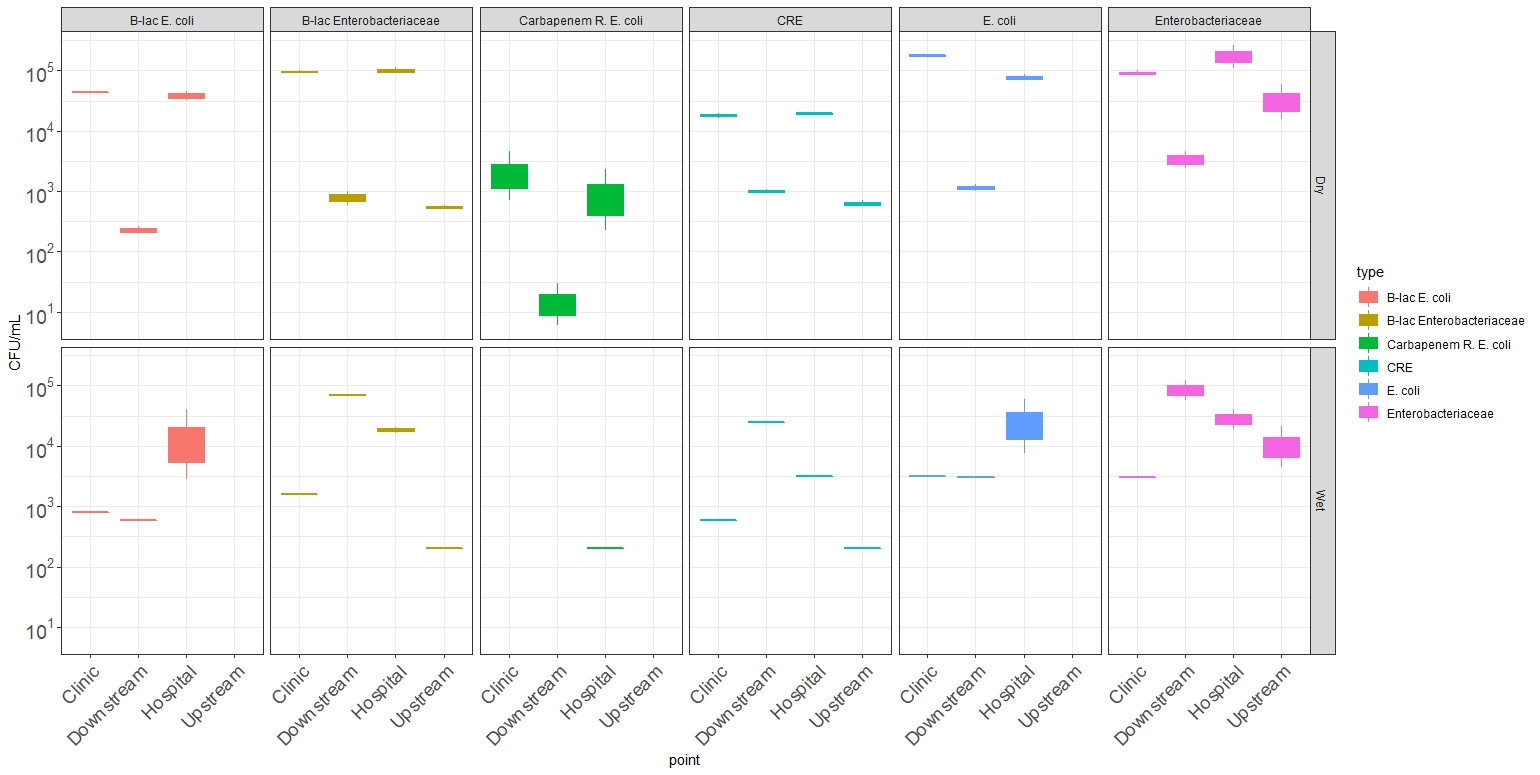


**Figure 2S.**


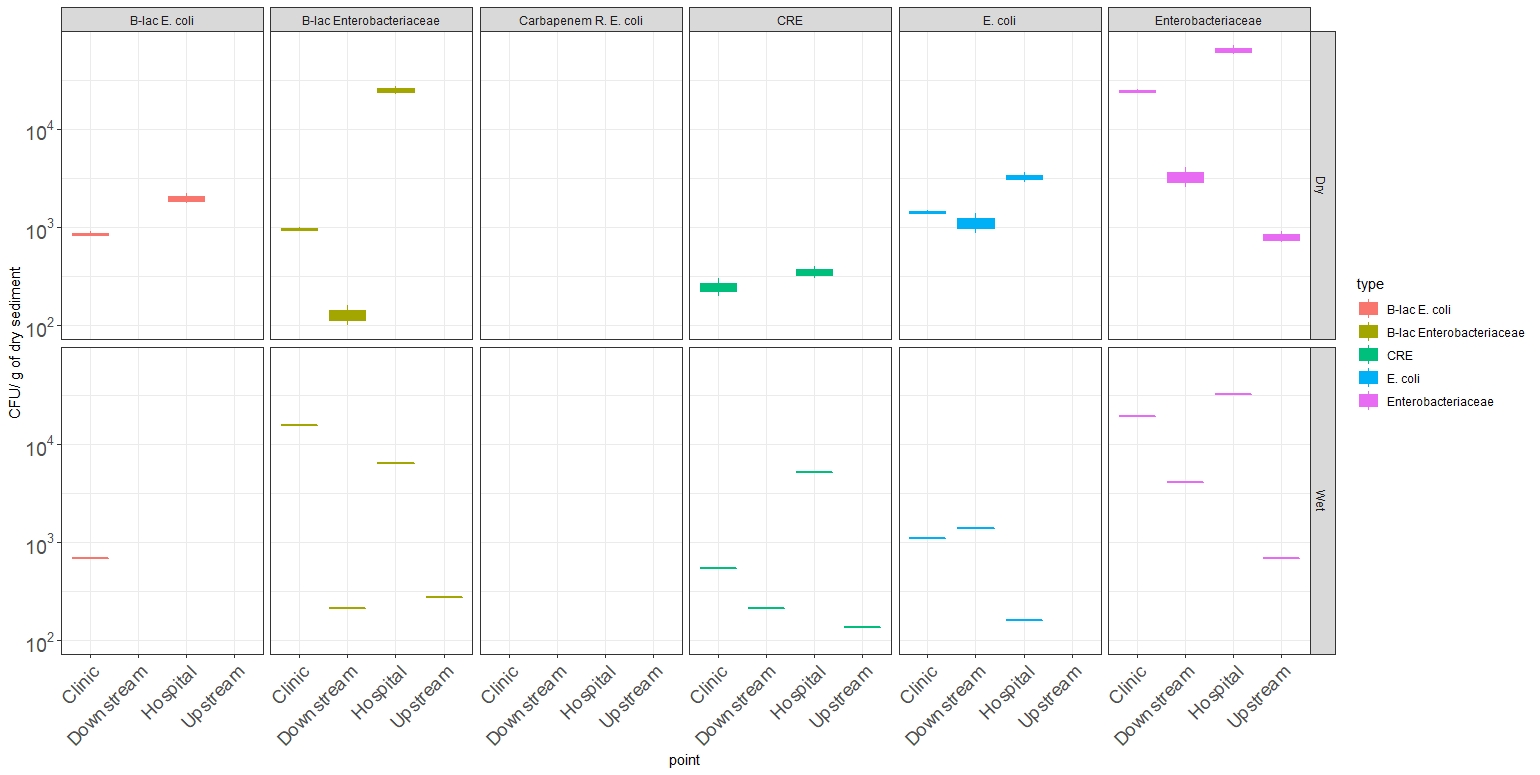


**Figure 3S.**


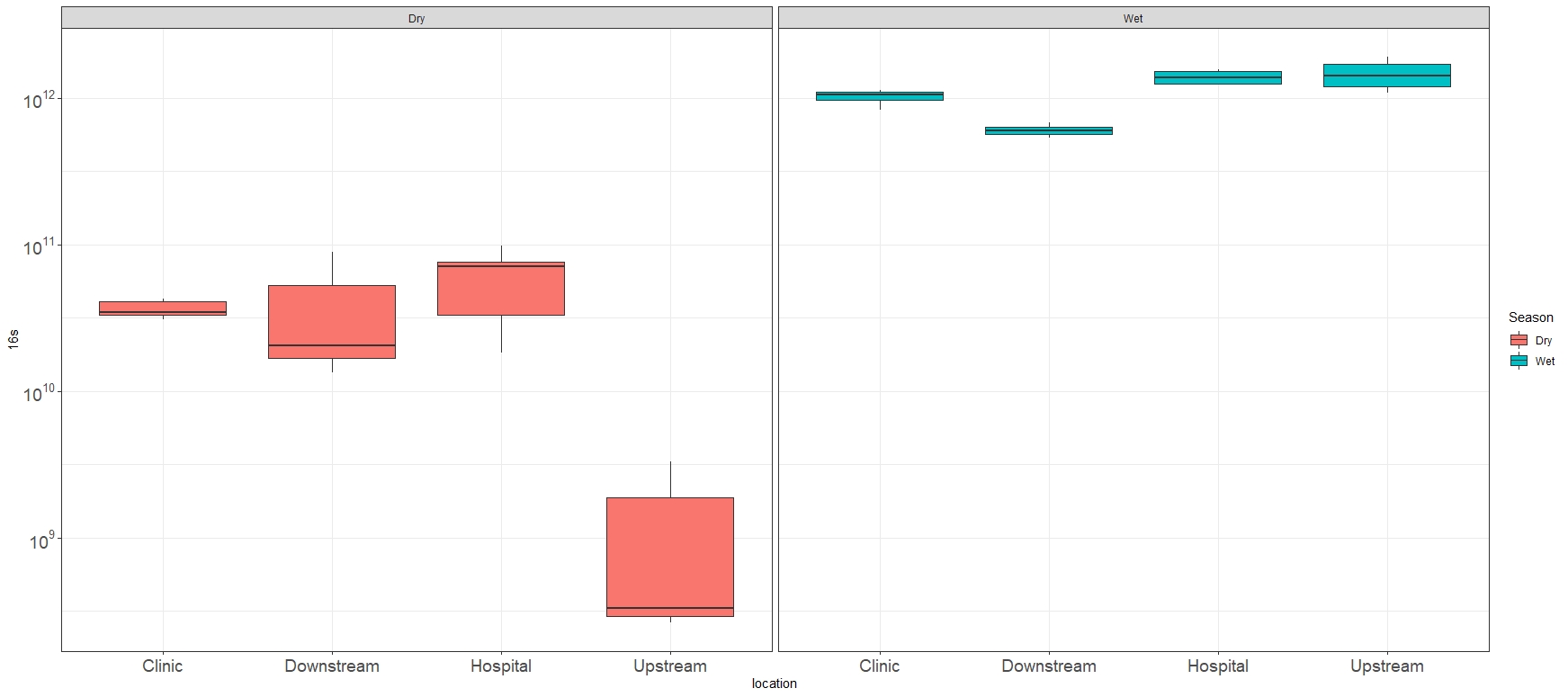


**Figure 4S.**


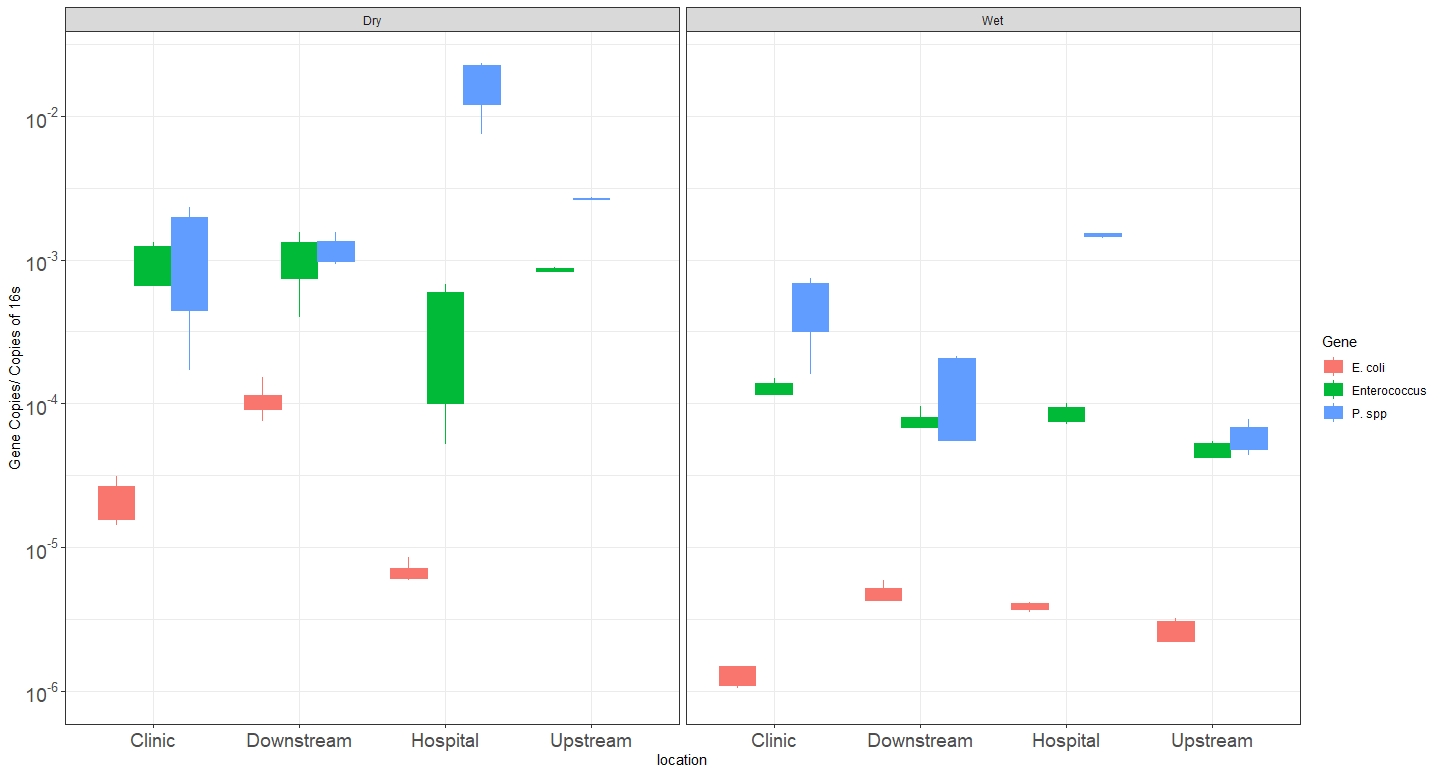


**Figure 5S.**


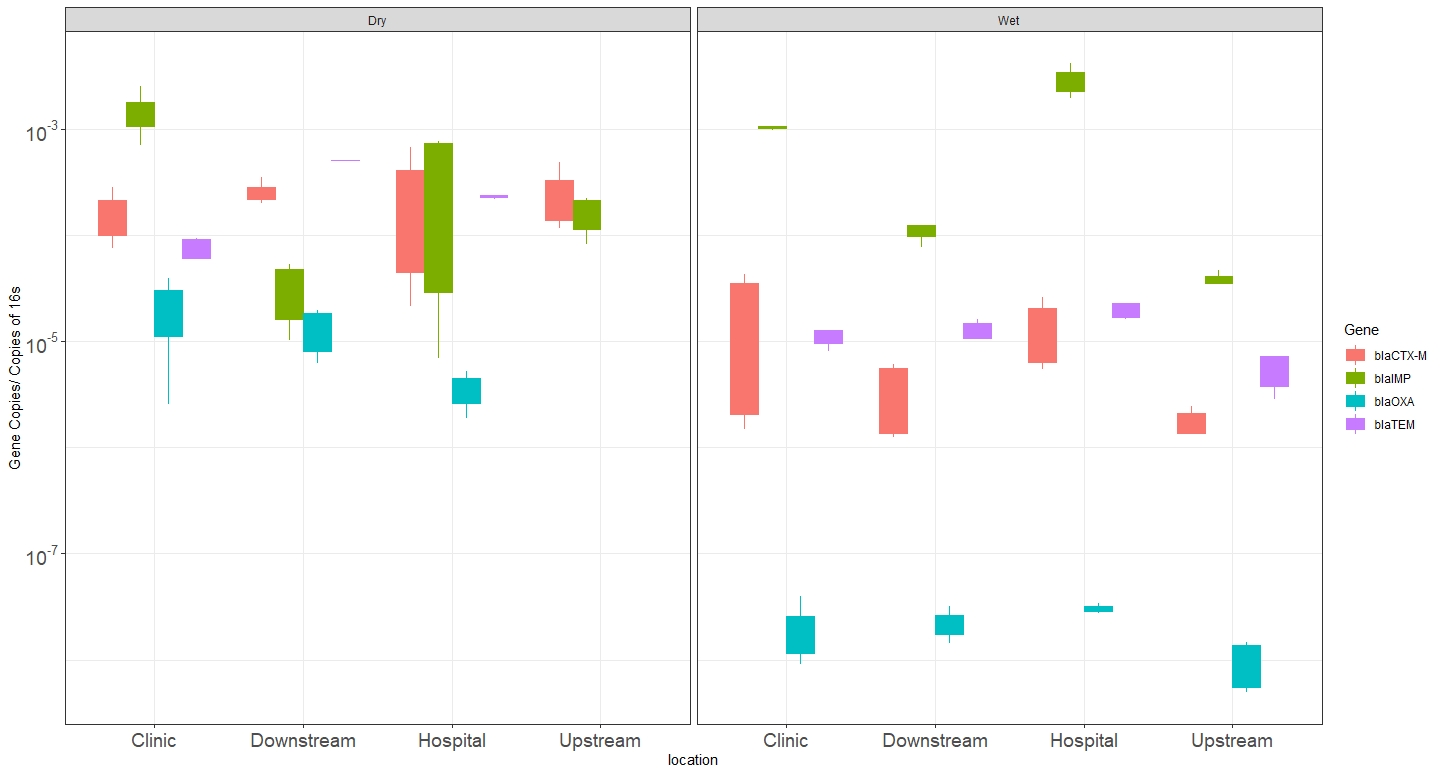


**Figure 6S.**

**
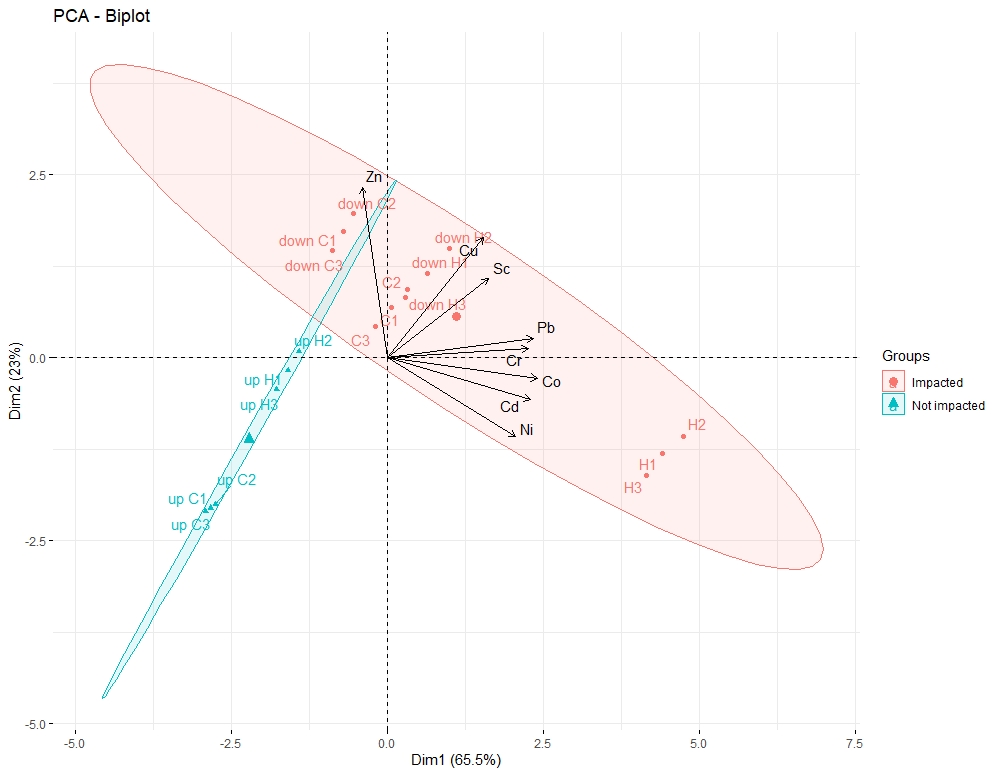
**

**References**

Bergmark, L., P. H. Poulsen, W. A. Al-Soud, A. Norman, L. H. Hansen, and S. J. Sorensen. 2012. 'Assessment of the specificity of Burkholderia and Pseudomonas qPCR assays for detection of these genera in soil using 454 pyrosequencing', *FEMS Microbiol Lett*, 333: 77-84.

Bisiklis, A., F. Papageorgiou, F. Frantzidou, and S. Alexiou-Daniel. 2007. 'Specific detection of blaVIM and blaIMP metallo-beta-lactamase genes in a single real-time PCR', *Clin Microbiol Infect*, 13: 1201-3.

Chern, E. C., S. Siefring, J. Paar, M. Doolittle, and R. A. Haugland. 2011. 'Comparison of quantitative PCR assays for Escherichia coli targeting ribosomal RNA and single copy genes', *Lett Appl Microbiol*, 52: 298-306.

Fujita, S., K. Yosizaki, T. Ogushi, K. Uechi, Y. Takemori, and Y. Senda. 2011. 'Rapid Identification of Gram-Negative Bacteria with and without CTX-M Extended-Spectrum beta-Lactamase from Positive Blood Culture Bottles by PCR Followed by Microchip Gel Electrophoresis', *Journal of Clinical Microbiology*, 49: 1483-88.

Ovreas, L., L. Forney, F. L. Daae, and V. Torsvik. 1997. 'Distribution of bacterioplankton in meromictic Lake Saelenvannet, as determined by denaturing gradient gel electrophoresis of PCR-amplified gene fragments coding for 16S rRNA', *Appl Environ Microbiol*, 63: 3367-73.

Poirel, L., T. R. Walsh, V. Cuvillier, and P. Nordmann. 2011. 'Multiplex PCR for detection of acquired carbapenemase genes', *Diagnostic Microbiology and Infectious Disease*, 70: 119-23.

Ryu, H., M. Henson, M. Elk, C. Toledo-Hernandez, J. Griffith, D. Blackwood, R. Noble, M. Gourmelon, S. Glassmeyer, and J. W. Santo Domingo. 2013. 'Development of quantitative PCR assays targeting the 16S rRNA genes of Enterococcus spp. and their application to the identification of enterococcus species in environmental samples', *Appl Environ Microbiol*, 79: 196-204.

Sidrach-Cardona, R., M. Hijosa-Valsero, E. Marti, J. L. Balcazar, and E. Becares. 2014. 'Prevalence of antibiotic-resistant fecal bacteria in a river impacted by both an antibiotic production plant and urban treated discharges', *Science of the Total Environment*, 488: 220-27.

1. * Corresponding author:

   John Poté, PhD.

   University of Geneva

   Faculty of Sciences

   Earth and Environmental Sciences

   Department F.-A. Forel

   Bd Carl-Vogt 66, CH-1211 Geneva 4

   Switzerland

   Tel: (+41 22) 379 03 21

   Fax: (+41 22) 379 03 29

   E-mail: [john.pote@unige.ch](mailto:john.pote@unige.ch) [↑](#footnote-ref-1)
